# Inverted Urothelial Papilloma in a Young Woman: A Case Report and Review of Diagnostic Advances

**DOI:** 10.7759/cureus.87287

**Published:** 2025-07-04

**Authors:** Ilias Kanakakis, Christos Giannoulis, Lazaros Tzelves, Dimitrios Vlachodimitropoulos, Marinos Berdempes

**Affiliations:** 1 Department of Urology, 417 Army Share Fund Hospital (NIMTS), Athens, GRC; 2 Second Department of Urology, National and Kapodistrian University of Athens, Sismanogleio General Hospital, Athens, GRC; 3 Department of Forensic Medicine and Toxicology, National and Kapodistrian University of Athens School of Medicine, Athens, GRC

**Keywords:** artificial intelligence, case report, differential diagnosis, immunohistochemistry, inverted urothelial papilloma, molecular biomarkers, radiomics, urothelial carcinoma

## Abstract

Inverted urothelial papilloma (IUP) is an uncommon benign urothelial tumor characterized by an endophytic growth pattern. While it most commonly affects older male patients, we report the case of a young female patient with a bladder lesion located on the right lateral wall, an exceptionally rare clinical presentation in terms of age, sex, and tumor location. Such unusual presentations offer valuable learning opportunities and remind clinicians to consider rare benign entities in the differential diagnosis of bladder tumors. Accurate differentiation from urothelial carcinoma (UC) remains essential due to overlapping histological features. We emphasize the critical role of imaging, histopathology, and immunohistochemistry in reaching a definitive diagnosis, while highlighting the potential of emerging methods to enhance clinical decision-making. As radiomics and artificial intelligence (AI) continue to advance, they may significantly influence diagnostic approaches and future therapeutic strategies.

## Introduction

Inverted urothelial papilloma (IUP) is an uncommon benign neoplasm of the urinary tract, first described by Paschkis in 1927 and subsequently named by Potts and Hirst in 1963 [1]. It predominantly arises in the bladder, especially in the neck and trigone regions, and demonstrates a marked male predominance with an approximate 6:1 male‑to‑female ratio [2].

Although IUP is histologically benign and accounts for <1 % of all urothelial tumors, its clinical significance stems from its close morphological resemblance to urothelial carcinoma (UC), complicating diagnostic accuracy and treatment decisions [3]. Ongoing developments in molecular profiling, immunohistochemistry (IHC), and artificial intelligence (AI)‑powered imaging analyses have markedly enhanced diagnostic precision, renewing focus on this understudied tumor entity.

Given the diagnostic challenges posed by IUP, a thorough understanding of its clinical, pathological, and molecular characteristics is essential for accurate differentiation from malignant urothelial lesions. In this context, we present an updated review of the current literature along with a rare case report, emphasizing the evolving role of emerging diagnostic technologies.

## Case presentation

**Figure 1:**
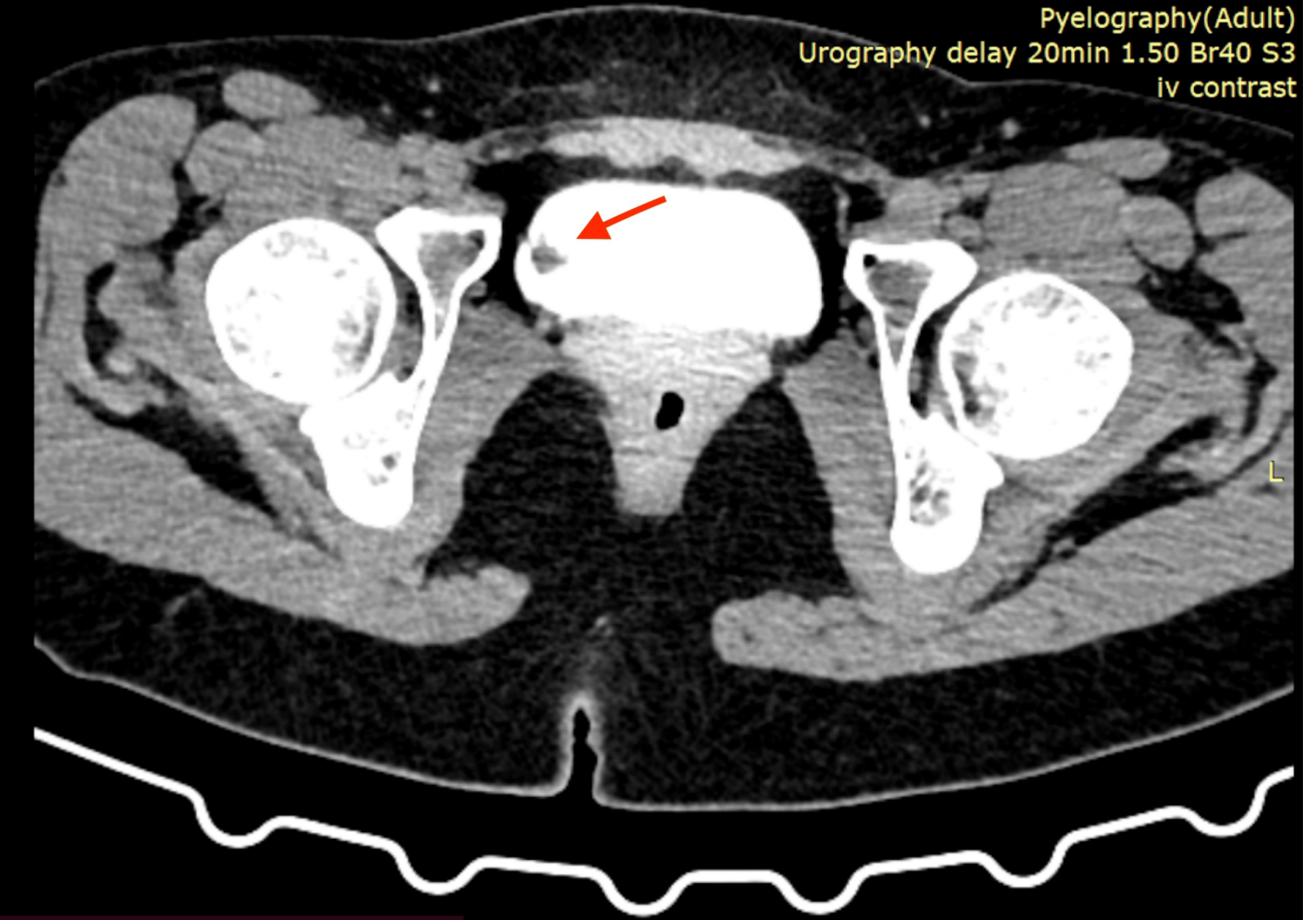
CTU showing a solitary papillomatous lesion at the right lateral bladder wall. CTU, computed tomography urography.

We present the case of a 23-year-old female patient who was evaluated for intermittent episodes of painless gross hematuria over two months. She had no significant past medical history and no history of smoking, occupational exposure to carcinogens, or urinary tract infections. Computed tomography urography (CTU) revealed a solitary, well-circumscribed papillomatous lesion on the right lateral wall of the bladder, measuring approximately 1.5 cm (Figure 1). 

**Figure 2:**
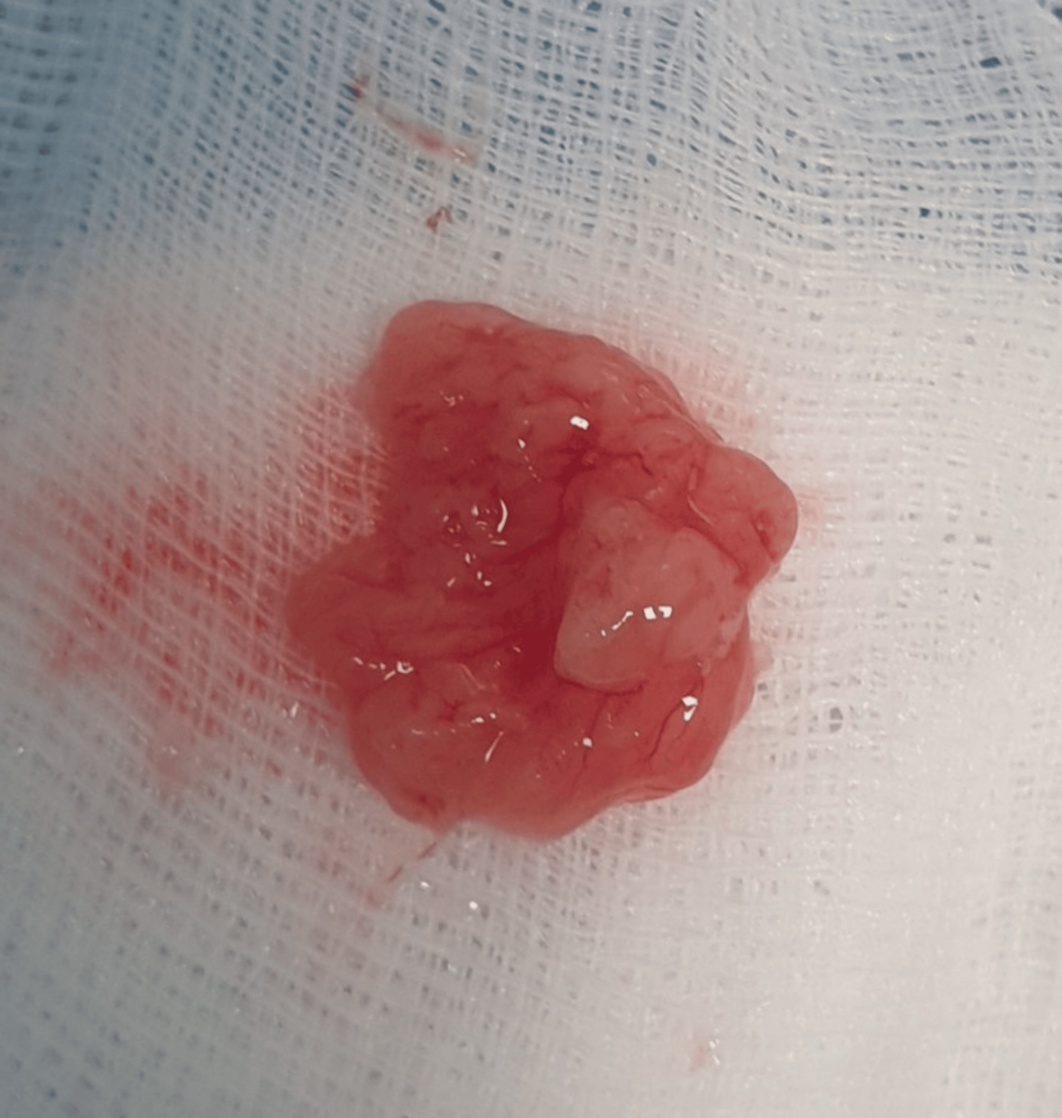
Surgical specimen after en bloc resection, showing a sessile polypoid mass consistent with inverted urothelial papilloma.

**Figure 3:**
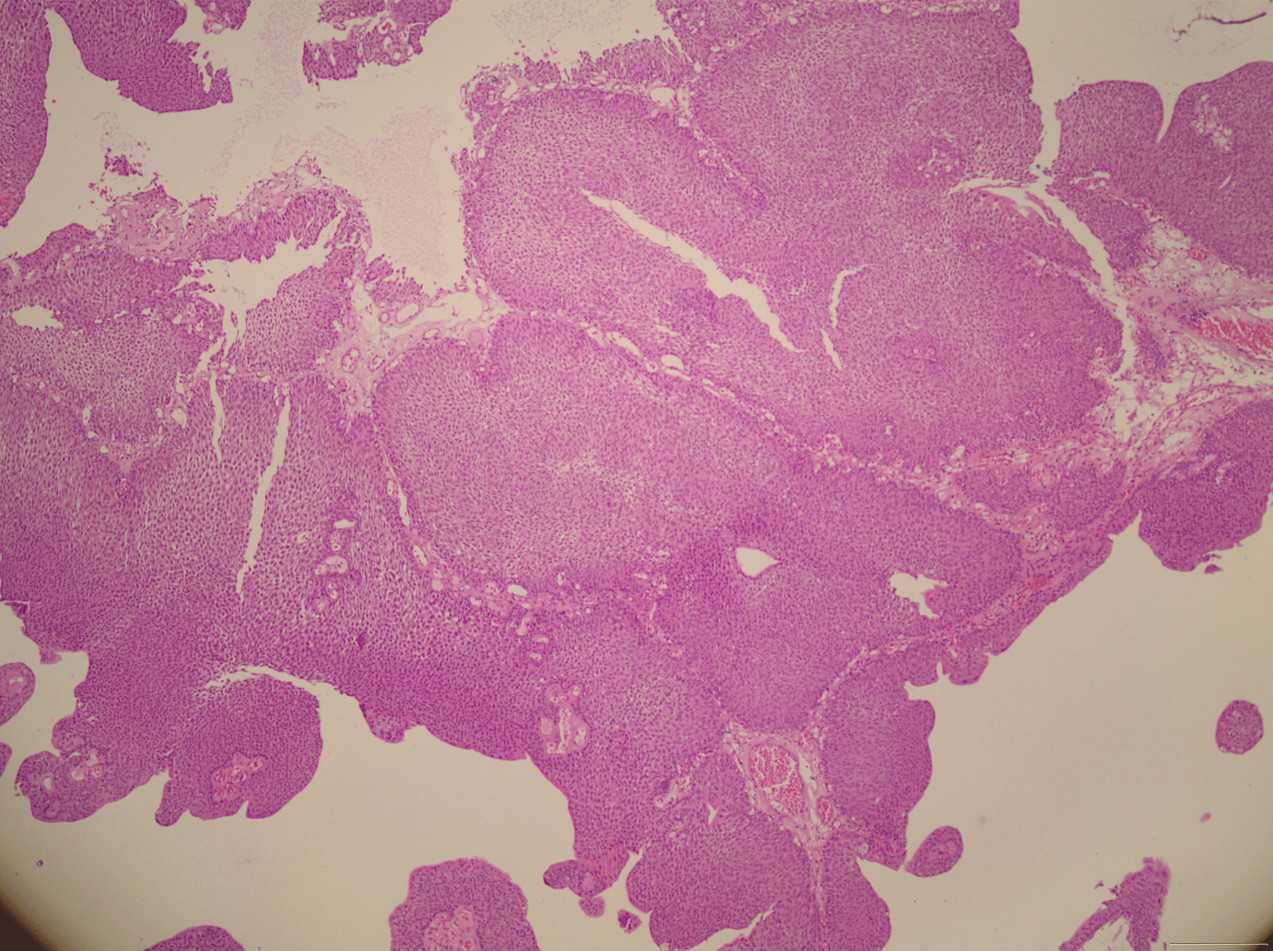
Histological section (H&E, ×40) showing endophytic growth of bland urothelial cells extending into the lamina propria, without atypia or mitoses, consistent with inverted urothelial papilloma.

The patient underwent transurethral resection of bladder tumor (TURBT). Gross examination revealed a sessile, grayish, friable, polypoid mass consistent with IUP (Figure 2). Histopathology confirmed an inverted growth of bland urothelial cells without atypia, mitoses, or necrosis (Figure 3). IHC showed a low Ki-67 index, focal CK20 positivity restricted to superficial layers, and absence of p53 overexpression, findings consistent with IUP. The patient experienced an uneventful recovery, with no postoperative complications or tumor recurrence during 12 months of cystoscopic surveillance. 

This case underscores the clinical relevance of accurate histopathological and immunohistochemical assessment, particularly in atypical demographics such as young women.

## Discussion

Etiology and pathogenesis

The etiology of IUP remains incompletely understood. Proposed contributing factors include chronic inflammation of the urothelium, exposure to occupational carcinogens (such as aromatic amines), smoking, and prior urinary tract infections [2]. Mechanical irritation from urinary catheters or stones has also been hypothesized as a potential trigger for urothelial proliferation. While environmental and lifestyle-related factors have been implicated, no definitive causal relationships have been established. Contemporary studies suggest that specific molecular alterations may play a role in its pathogenesis, although the precise mechanisms remain to be fully elucidated.

Clinical presentation

IUP is most often asymptomatic and frequently discovered incidentally during evaluation for unrelated urinary symptoms. When present, gross or microscopic hematuria is the most common clinical manifestation, typically painless in nature. Less commonly, patients may experience irritative voiding symptoms such as dysuria, flank pain, or urinary obstruction, depending on the tumor's size and anatomical location [4]. Notably, the clinical presentation of IUP is non-specific and can mimic that of UC or other bladder lesions. Moreover, although IUP predominantly affects middle-aged males, rare cases, such as our reported young female patient, highlight the necessity for maintaining a broad differential diagnosis.

Imaging and endoscopic findings

On cystoscopy, IUPs typically present as solitary, smooth-surfaced, pedunculated or sessile lesions [5]. CTU often reveals a well-circumscribed, homogeneously enhancing intraluminal mass without evidence of muscular invasion, perivesical fat stranding, or regional lymphadenopathy. Radiologically, IUPs demonstrate smooth margins and uniform enhancement [6], in contrast to the irregular, heterogeneous, and infiltrative patterns observed in UC (Table 1). Recognizing these distinct imaging features may aid in preoperative suspicion of a benign lesion, although definitive histopathological confirmation remains essential.

**Table 1:** Comparative clinicopathological and imaging features of IUP and UC. IUP, inverted urothelial papilloma; UC, urothelial carcinoma; FGFR3, fibroblast growth factor receptor 3; TERT, telomerase reverse transcriptase; TP53, tumor protein p53. Source: Refs. [2,7-9].

Feature	ΙUP	UC
Growth pattern	Endophytic, smooth, well-circumscribed nests	Infiltrative, irregular, poorly demarcated nests
Cytological atypia	Minimal or absent	Pronounced
Mitoses	Rare	Frequent
Necrosis	Absent	Common
Ki-67 proliferation index	Low (<1%)	High
p53 overexpression	Rare	Frequent
PYGB expression	Ηigh	Low
Molecular profile	HRAS mutations; absence of TERT promoter mutations	FGFR3, TP53, and TERT promoter mutations
Imaging characteristics	Smooth margins, homogeneous enhancement	Irregular margins, heterogeneous enhancement

Histopathology and IHC

IUP is characterized histologically by an endophytic (inverted) growth pattern of uniform, cytologically bland urothelial cells extending into the lamina propria without evidence of architectural disarray, cytological atypia, or significant mitotic activity [3]. Necrosis is notably absent (Table 1).

IHC typically demonstrates a low Ki-67 proliferation index (<1%), focal CK20 expression confined to the superficial urothelial layers, and rare or absent p53 overexpression. These immunoprofiles are instrumental in differentiating IUP from UC, which demonstrates high Ki-67, diffuse CK20 staining, and frequent p53 mutations [7,10].

Molecular characteristics

Recent molecular studies have provided critical insights into the biological distinction between IUP and UC. IUPs frequently harbor activating mutations in the HRAS gene, a feature that is notably absent in conventional UC [11]. Conversely, fibroblast growth factor receptor 3 (FGFR3) mutations, telomerase reverse transcriptase (TERT) promoter mutations, and tumor protein p53 (TP53) alterations are commonly detected in UC but are rare or absent in IUP. Particularly, the absence of TERT promoter mutations in IUP holds significant diagnostic relevance, as TERT alterations are a hallmark of malignancy in urothelial tumors.

In addition, recent proteomic analyses, including machine learning-based approaches, have identified brain glycogen phosphorylase (PYGB) as a novel biomarker with significantly higher expression in IUP compared to UC [8]. This differential expression suggests that PYGB may serve as an additional diagnostic adjunct in challenging cases, potentially enhancing the specificity of histopathological evaluation (Table 1).

AI in differentiating IUP from UC

Technological developments in AI and radiomics have significantly transformed the diagnostic landscape of bladder tumors [12]. Radiomics, which involves the extraction of high-dimensional quantitative features from medical images, enables the capture of subtle imaging patterns that may escape human visual perception. Machine learning algorithms trained on radiomic features derived from CTU have demonstrated excellent performance in differentiating IUP from UC [9,13].

Growing literature has highlighted the increasing importance of AI in enhancing bladder cancer diagnostics, outlining both current applications and future potential. Moreover, machine learning approaches have also been applied to proteomic data, showing promise in the identification of novel diagnostic biomarkers that could complement traditional histopathology [14].

Management and prognosis

The treatment of choice for IUP is complete TURBT [5]. Following resection, the risk of recurrence is low, reported between 1% and 7% [4]. Nevertheless, follow-up cystoscopic surveillance is recommended due to the potential, albeit rare, for local recurrence or initial misclassification of the lesion. Current practice suggests cystoscopy every three to six months during the first year, followed by annual examinations thereafter if no recurrence is observed [2]. No cases of metastasis or malignant transformation have been documented. Accurate diagnosis and complete resection confer an excellent prognosis.

## Conclusions

IUP is a rare, benign neoplasm with distinctive histological and molecular characteristics that allow reliable differentiation from UC. Accurate diagnosis is critical to avoid unnecessary radical treatments and to ensure optimal patient outcomes. Recent advances in AI, radiomics-based imaging analysis, and molecular and immunohistochemical markers have introduced promising non-invasive strategies for improving diagnostic precision and reducing misclassification.

In our case, while conventional imaging and histopathology successfully established the diagnosis of IUP, emerging AI-driven radiomics models could have suggested the benign nature of the lesion preoperatively, potentially reducing diagnostic uncertainty. However, surgical resection via transurethral resection of the bladder tumor would still have been necessary to obtain definitive histopathological confirmation and to guide appropriate management decisions. Future studies should aim to validate the clinical utility and diagnostic performance of AI-driven models and molecular biomarkers, not only in the evaluation and differentiation of urothelial tumors, but also in guiding future management strategies.
